# The Public Services

**Published:** 1918-09-07

**Authors:** 


### THE PUBLIC SERVICES.

### ROYAL NAVAL MEDICAL SERVICE.

*Qualification.*—1. Every candidate for admission into the Medical Department of the Royal Navy must be not under 21 nor over 28 years of age on the day of the commencement of the competitive examination. He must produce an extract from the register of the date of his birth, or, in default, a declaration made before a magistrate stating the date of birth.

2. He must declare: (1) His age and date and place of birth; (2) that he is of pure European descent, and the son of natural-born British subjects; (3) that he labours under no mental or constitutional disease or weakness, nor any other imperfection or disability which may interfere with the most efficient discharge of the duties of a medical officer in any climate; (4) that he is ready to engage for general service at home or abroad if required; (5) whether he holds, or has held, any commission or appointment in the Public Services; (6) that he is registered under the Medical Act, giving the date of his registration as a medical student or of his beginning professional study; (7) whether he has been previously examined for entry into the Naval Service, and, if so, when.

3. The certificates of registration and birth must accompany the declaration, which is to be filled up and returned as soon as possible, addressed to the Director-General, Medical Department, Admiralty, S.W., before the candidate can be allowed to compete for an existing vacancy.

4. The candidate will be examined as to his physical fitness by a Board of Naval Medical Officers.

5. Candidates who have served in the Officers' Training Corps will be credited with additional marks at the entrance examination in the following proportion: Those in possession of Certificate A, 1 per cent.; those in possession of Certificates A and B, 2 per cent. of the maximum number of marks allotted.

*Examination.*—1. Entrance examination held twice yearly in London in the following subjects: (*a*) Medicine, including medical pathology and therapeutics; (*b*) surgery, including surgical pathology and clinical surgery.

No candidate will be considered eligible unless he obtains 50 per cent. of the marks in each subject.

Successful candidates will be given appointments as acting surgeons in the Royal Navy, and will then enter upon a six months' course of training.

The first two months will be passed at the Naval Medical School, Greenwich. Here students will receive instruction in tropical medicine, pathology, and hygiene. The remaining four months will be passed at Haslar, and will be devoted to the study of naval hygiene, recruiting, physical training, diving and submarine work, radiography, anæsthetics, dentistry, etc.

*Promotions.*—Promotion to staff-surgeons and fleet-surgeons takes place at the end of eight and sixteen years respectively—provided the qualifying examination for staff-surgeon has been passed. In addition, special promotion will be made at their Lordships' discretion as a reward for distinguished service or conspicuous professional merit.

Accelerated promotion will be granted to officers who at the qualifying examination for staff-surgeon obtain at least 75 per cent. of the total marks.

Promotion to the ranks of deputy surgeon-general and surgeon-general are made by selection from the ranks of fleet-surgeons and deputy surgeons-general respectively.

*Withdrawals.*—Officers will be allowed to withdraw, at the discretion of their Lordships, with gratuities on the following scale: After four years' service, £500; after eight years' service, £1,000; after twelve years' service, £1,500; and after sixteen years' service, £2,250.

Entries into the Navy during the war for permanent service are suspended.

### ROYAL ARMY MEDICAL CORPS.

A candidate for a commission in the Royal Army Medical Corps must fulfil certain physical and medical requirements, and must be 21 years and not over 28 years of age at the date of the commencement of the Entrance examination. He must be registered under the Medical Acts in force in the United Kingdom. Before being permitted to proceed to the Entrance examination the candidate is required to attend at the War Office about two days before the commencement for the purpose of being interviewed and undergoing physical examination. The Entrance examination is held twice a year in London—January and July—a fee of £1 being charged for admission thereto. It lasts about three days. The examination is of a clinical, written, practical, and oral character. It consists of: A commentary on a case in medicine; an essay in surgery; examination and report on a clinical medical case; examination and report on clinical surgical cases; orals in clinical medicine, clinical surgery, and in medical and surgical pathology; also surgical operations and bandaging (including surgical instruments and appliances). A candidate who is successful at the Entrance examination is appointed a lieutenant (on probation), and is then required to attend at the Royal

Army Medical College, London, and afterwards at the Royal Army Medical Corps School of Instruction, for the purpose of qualifying in the following subjects before his commission can be confirmed :—Royal Army Medical College : Military surgery, tropical medicine, hygiene, pathology, and organisation of military hospitals, and principles governing medical charge of troops. Royal Army Medical Corps School of Instruction : Regimental duties, drill and field training.

Further particulars relating to marks given for service in the O.T.C. or T.F., etc., and regarding the holding of civil appointments after having passed the Entrance examination are contained in "Regulations for Admission to the R.A.M.C.," which can be obtained on application to the Secretary, War Office.

The Entrance examination is in abeyance at the present time.

### INDIAN MEDICAL SERVICE.

1. Since the open competitive examination held in July 1915 for admission to the Indian Medical Service no similar examination has been held during the war, but such appointments as are required to meet the absolutely indispensable needs of the Service have been made by nomination by the Secretary of State. To assist him in making these appointments, which are limited in number to the absolutely indispensable needs of the Service, the Secretary of State for India has appointed a Selection Committee who will summon and interview such applicants as may appear to be *prima facie* suitable, and make recommendations for appointment.

Applications for appointment should be addressed to the Secretary, Military Department, India Office, Whitehall, London, S.W., 1, and should contain concise particulars of the applicant's medical degrees and career. Applicants must be over twenty-one and under thirty-two years of age at the time of application. Particulars regarding pay, promotion, etc., in the Service can be obtained from the Military Department, India Office.

2. *Procedure for appointment in force at the last Competitive Examination.*—(1) The Regulations are those in force at the present time. They are subject to any alterations that may be determined on; (2) Every candidate must be a British subject of European or East Indian descent, and his father must at the time of the candidate's birth have been either a British subject born within His Majesty's Allegiance, or a person to whom a certificate of naturalisation had been granted, or a subject of a State in India under the suzerainty of His Majesty; and such father must still be, or have continued to be till his death, a British subject or a subject of such a State in India.

Provided that a subject of any State in India in respect of whom the Governor-General in Council has made a declaration under Section 96*a* of the Government of India Act, 1915, as amended by Section 3 of the Government of India (Amendment) Act, 1916, may be considered eligible.

Every candidate must also be of sound bodily health, and, in the opinion of the Secretary of State for India in Council, in all respects suitable to hold a commission in the Indian Medical Service. He may be married or unmarried. He must possess a qualification registrable in Great Britain and Ireland under the Medical Acts in force at the time of his appointment.

No candidate will be permitted to compete more than three times. Candidates must be between 21 and 28 years of age on the first day of the month following that in which the examination is held. But see note 1 above.

The physical fitness of each candidate will be determined by a Board of Medical Officers, who are required to certify that his vision is sufficiently good to enable him to pass the tests laid down by the Regulations. The candidate will be examined by the Examining Board in the following subjects : (1) Medicine, including therapeutics; (2) surgery, including diseases of the eye; (3) applied anatomy and physiology; (4) pathology and bacteriology; (5) midwifery and diseases of women and children; (6) materia medica, pharmacology, and toxicology. The examination in medicine and surgery will be in part practical, and will include operations on the dead body, the application of surgical apparatus, and the examination of medical and surgical patients at the bedside. No syllabus is issued in the subjects of the examination, which will be conducted so as to test the general knowledge of the candidate in these subjects. No candidate shall be considered eligible who shall not have obtained at least one-third of the marks obtainable in each of the above subjects and one-half of the aggregate marks for all the subjects.

After passing the competitive examination, the successful candidates will be required to attend a course of practical instruction at the Army Medical School and elsewhere, as may be decided, in : (1) hygiene; (2) military and tropical medicine; (3) military surgery; (4) pathology of diseases and injuries incidental to military and tropical service. This course will be of not less than four months' duration, and at its conclusion candidates will be required to pass an examination in the subjects taught therein.

3. After the war the Secretary of State will make further appointments to the Service from among duly qualified persons, European and Indian, who have held temporary commissions in the Indian Medical Service or the Royal Army Medical Corps during the war, and have served with the British or Indian Expeditionary Forces or hospitals and hospital ships for soldiers. The date of the resumption of competitive examinations, and the conditions of such examinations, will be announced in due course.

4. Full particulars regarding the Service generally can be obtained by application to the Secretary, Military Department, India Office, London, S.W. 1.

### COLONIAL MEDICAL SERVICE.

All applicants must be between the ages of twenty-three and thirty-five, but in the case of East Africa, Uganda, Nyassaland, Somaliland, and Zanzibar, preference is given to candidates over twenty-five, and in the case of Fiji and the Western Pacific to candidates under thirty. Candidates must be doubly qualified, and those who have previously held hospital appointments are preferred. Before appointment they will have to submit to medical examination by one of the consulting physicians of the Colonial Office.

In the majority of instances the duties required of a Colonial medical officer are of a general character. They include medical, surgical, and not infrequently public health work. Occasionally only is a specialist required.

Full details regarding this Service and the advantages it offers to newly qualified men may be obtained on application to the Private Secretary, Colonial Office, S.W.

